# Placental RNA sequencing implicates IGFBP1 in insulin sensitivity during pregnancy and in gestational diabetes

**DOI:** 10.21203/rs.3.rs-3464151/v1

**Published:** 2023-10-27

**Authors:** Marie-France Hivert, Frederique White, Catherine Allard, Kaitlyn James, Sana Majid, François Aguet, Kristin Ardlie, Andrea Edlow, Jose Florez, Luigi Bouchard, Pierre-Etienne Jacques, S. Karumanchi, Camille Powe

**Affiliations:** Harvard Medical School; Universite de Sherbrooke; Universite de Sherbrooke; Massachusetts General Hospital; Harvard Medical School; Broad Insitute of MIT and Harvard; Broad Institute of MIT & Harvard; Massachusetts General Hospital and Harvard Medical School; Massachusetts General Hospital; Department of Biochemistry, Université de Sherbrooke/ECOGENE-21 and Lipid Clinic, Chicoutimi Hospital; Universite de Sherbrooke; Cedars-Sinai Medical Center; Diabetes Unit, Division of Endocrinology, Massachusetts General Hospital, Boston, MA.

**Keywords:** transcriptomics, placenta, insulin sensitivity, pregnancy, RNA sequencing, Insulin Growth Factors (IGF), prediction, gestational diabetes mellitus

## Abstract

Reduced insulin sensitivity (or greater insulin resistance) is a hallmark of normal physiology in late pregnancy and also underlies gestational diabetes mellitus (GDM) pathophysiology. We conducted transcriptomic profiling of 434 human placentas and identified a strong positive association between insulin-like growth factor binding protein 1 gene (*IGFBP1*) expression in the placenta and insulin sensitivity at ~ 26 weeks’ gestation. Circulating IGFBP1 protein levels rose over the course of pregnancy and declined postpartum, which together with high placental gene expression levels, suggests a placental source. Higher circulating IGFBP1 levels were strongly associated with greater insulin sensitivity (lesser insulin resistance) at ~ 26 weeks’ gestation in the same cohort and two additional pregnancy cohorts. In addition, low circulating IGFBP1 levels in early pregnancy predicted subsequent GDM diagnosis in two cohorts. These results implicate IGFBP1 in the glycemic physiology of pregnancy and suggest a role for placental IGFBP1 deficiency in GDM pathogenesis.

## Introduction

Gestational diabetes mellitus (GDM) affects 1 in 7 pregnancies worldwide^1^ and is associated with maternal and offspring adverse health outcomes during pregnancy, at delivery, and over the life course^2^. Prior research has established that a defect in insulin sensitivity (i.e., greater insulin resistance) contributes to GDM^3,4^. In addition, we and others^5–10^ have previously shown that among individuals with GDM, those with the lowest insulin sensitivity (insulin resistant GDM) have the greatest risk of hyperglycemia-associated pregnancy complications, suggesting that reduced insulin sensitivity is a key contributor not only to GDM itself, but also to the negative health outcomes that accompany it.

The placenta is the major driver of marked changes in insulin physiology during pregnancy, including the drastic decline in insulin sensitivity, which occurs even in those without GDM. This has been attributed to hormonal factors released by the placenta that lead to insulin resistance^11^. Yet, the specific placental circulating factors that mediate the profound change in insulin sensitivity during pregnancy are still unknown, and the classically-implicated pregnancy hormones (e.g., human chorionic gonadotropin, human placental lactogen, and placental growth hormone) are poorly correlated with insulin sensitivity in pregnancy^12^. Better understanding of the placental factors driving the pregnancy-related decline in insulin sensitivity could lead to novel therapeutic approaches to hyperglycemia, early identification of those at risk of developing GDM, and recognition of those most likely to have GDM-related pregnancy complications. Despite the hallmark reduction in insulin sensitivity in all pregnancies, most pregnant individuals do not develop GDM. This phenomenon suggests that additional factors, yet unknown, may contribute to the maintenance of euglycemia in pregnancy. Indeed, a variable improvement in insulin sensitivity has been reported in early pregnancy in several studies^13,14^. A systematic search for placental factors that are related to insulin sensitivity in pregnancy may also uncover those that improve it.

The overarching goal of this study was to discover novel placental factors implicated in physiologic changes in insulin sensitivity during pregnancy and that contribute to GDM pathophysiology. We conducted a placental genome-wide transcriptomic study using RNA sequencing (RNA-seq) to identify genes whose expression in the placenta was associated with insulin sensitivity in pregnancy. We identified *IGFBP1* as the most strongly associated placental transcript. We then measured circulating IGFBP1 protein in plasma samples collected from three different pregnancy cohorts, at multiple time-points during and after gestation. Using these data, we investigated associations between circulating IGFBP1 levels and insulin sensitivity, other pregnancy-related metabolic traits, birth anthropometric measurements, and risk of GDM.

## Results

### Participants included in placental genome-wide RNA sequencing analyses

We conducted a genome-wide RNA-seq study using placental samples collected from 434 participants in the Genetic of Glucose regulation in Gestation and Growth (Gen3G) prospective pregnancy cohort^15^ (Table 1). At study entry (median: 9 weeks’ gestation), participants’ mean ± SD age was 28.7 ± 4.4 years, and median [IQR] body mass index (BMI) was 23.8 [21.4–27.9] kg/m^2^. We excluded individuals with diabetes present prior to pregnancy. Participants underwent a fasting 75g oral glucose tolerance (75g-OGTT) in the late second trimester (median: 26 weeks’ gestation), during which we collected plasma samples and measured glucose and insulin levels at multiple time-points to estimate insulin sensitivity (using the Matsuda index, which has been previously validated against euglycemic clamps in pregnancy^16^). At delivery (median [IQR] = 39.6 [38.7–40.3] weeks), we collected samples from the maternal-facing side of the placenta using standardized protocols for collection and storage for future analyses by RNA-seq (see Methods).

### Differential placenta RNA expression in relation to insulin sensitivity in pregnancy

**Figure 1:**
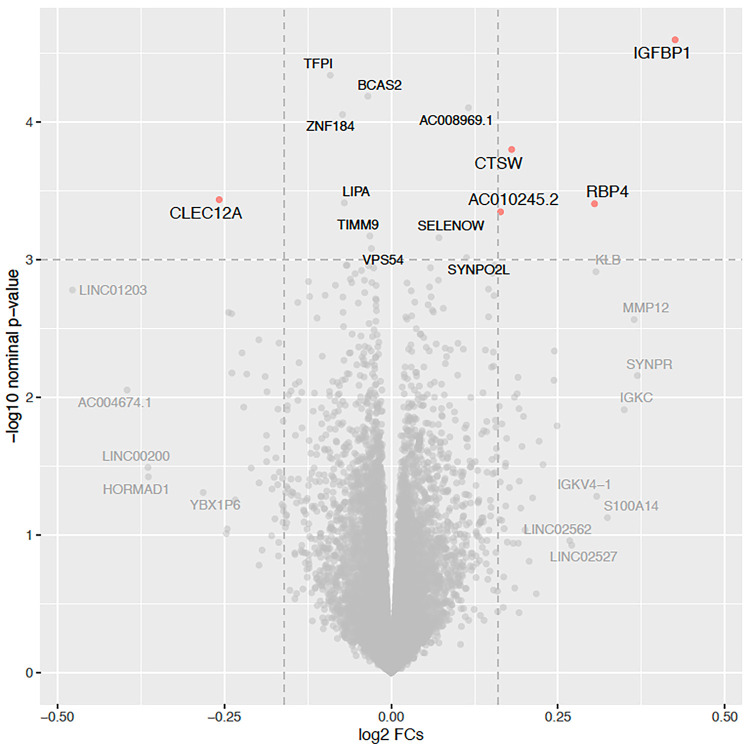
Volcano plot showing placenta RNA differential expression in relation to insulin sensitivity (Matsuda, log2 transformed) at 26 weeks’ gestation in 434 Gen3G participants model adjusted for maternal age, gravidity, maternal BMI at first trimester visit, sex of offspring, and gestational age at delivery, and 37 SVs (from SmartSVA package); gene names identified if *P*-values <1.0 × 10^−3^

After processing and quality control of the placental RNA-seq dataset, we investigated differential expression of 15,202 genes in relation to insulin sensitivity (Matsuda index, log2 transformed) in late second trimester. We identified 14 genes whose placental RNA expression levels were associated with insulin sensitivity (*P*-values < 1.0×10^−3^; Supp Table 1) after accounting for technical variability (37 Surrogate variables (SV)), precision variables (gestational age at delivery, fetal sex), and potential confounders (gravidity, maternal age, and BMI) using multivariate linear regression models. We observed the strongest association between insulin sensitivity and *insulin-like growth factor binding protein 1* gene (*IGFBP1*; β = 0.43; *P* = 2.5×10^−5^), where higher placental expression levels were associated with greater insulin sensitivity (Fig. 1). IGFBP1 is a binding protein that is primarily produced by the liver outside of pregnancy and is highly expressed by the placenta^17^ (https://www.proteinatlas.org/ENSG00000146678-IGFBP1), as replicated in our RNA-seq data. IGFBP1 has been implicated in the modulation of the biological activity of IGF-1 and IGF-2, which are key regulators of growth and metabolism in post-natal and fetal life^18^. We did not observe strong associations between the Matsuda index and other genes in IGF-related pathways, or genes encoding classic pregnancy-specific placental hormones, or genes encoding inflammatory proteins secreted by the placenta that have been previously associated with insulin sensitivity in pregnancy^12^ (Supp. Table 2).

### Physiology of circulating IGFBP1 levels during pregnancy and gestational OGTT

Given the high levels of placental expression of *IGFBP1* (average TPM = 103.4) and its known secreted protein status, we measured circulating levels of IGFBP1 (R&D systems ELISA #DGB100) in Gen3G participants (n = 837; Supp. Table 3) and two additional pregnancy cohorts: the Study of Pregnancy Regulation of Insulin and Glucose (SPRING) and the MGH Obstetrical Maternal Study (MOMS). Both SPRING and MOMS are diverse cohorts based in the US, and SPRING has enrichment for individuals at high risk of developing GDM based on established clinical factors (Supp. Table 4). SPRING was also designed to characterize the physiology of glycemic and insulin regulation longitudinally during and after pregnancy^19^.

In SPRING participants who remained normoglycemic throughout pregnancy (n = 65), we observed that median plasma levels of IGFBP1 rose between the 1st trimester (66,610 pg/mL) and 24 to 32 weeks’ gestation (79,379 pg/mL), then declined dramatically post-partum (16,588 pg/mL; paired t-tests *P* < 0.001 for differences between plasma levels across pregnancy and postpartum; Supp. Figure 1). This pattern, combined with high placental expression levels, suggests a placental origin of high circulating IGFBP1 levels during pregnancy.

In a subset of Gen3G participants (n = 27) in whom we assayed serial IGFBP1 levels during the 75g-OGTT (Supp. Figure 2), we observed that circulating IGFPB1 levels were stable over the first hour of the OGTT (median levels: fasting = 87,008 pg/mL; 1h-post load = 91,485 pg/mL; paired t-test *P*-value = 0.13), but declined 2h-post glucose load (median = 60,920 pg/mL; paired t-test *P*-value = 0.0007 compared to fasting). The change in plasma insulin levels from baseline to 1h (delta insulin 0–60 minutes) appeared to be inversely associated with the IGFBP1 levels at 1h (r= −0.39; *P* = 0.047) and at 2h (r= −0.31; *P* = 0.11) during the OGTT. This is consistent with the known negative feedback regulation of IGFBP1 expression by insulin, albeit only shown in hepatocytes^20^.

### Circulating IGFBP1 is correlated with insulin sensitivity in three independent pregnancy cohorts

Higher plasma IGFBP1 levels were associated with greater insulin sensitivity in all three pregnancy cohorts examined (Table 2). The strong positive correlations (Pearson r = 0.5 to 0.6; *P* < 0.001) between plasma IGFBP1 levels and insulin sensitivity were consistent across different periods of pregnancy, as well as in the postpartum period (SPRING). Adjusting for maternal age and gestational age at the time of blood sampling did not influence correlations. The strength of association was modestly attenuated by further adjustment for maternal BMI, but it remained highly statistically significant (r = 0.34 to 0.48, *P* < 0.001; Table 2).

In Gen3G, we further assessed Pearson correlations between plasma IGFBP1 (in the first and second trimester) and various maternal metabolic traits and neonatal anthropometric measures (Supp. Table 5). Higher maternal BMI was associated with lower plasma IGFBP1 in the first trimester (r= −0.27) and in the late second trimester (r= −0.54; both *P* < 0.001). Plasma IGFBP1 in the late second trimester was negatively correlated with glucose (r= −0.28 to −0.30) and insulin levels (r= −0.40) during the OGTT (all *P* < 0.001). Lower IGFBP1 levels at both time-points were also associated with higher birth weight z-scores (standardized for gestational age and sex) at delivery (r= −0.15 and r= −0.21 for IGFBP1 at the first and second trimester visits, respectively; both *P* < 0.001). Adjusting for maternal BMI or for maternal glucose reduced the strength of associations, but the correlations remained statistically significant (e.g. second trimester IGFBP1 partial correlations with birth weight Z-score adjusted for maternal BMI r= −0.12; *P* < 0.001; or adjusted for maternal glucose (glucose area under the curve (AUC) during the OGTT) r= −0.17; *P* < 0.001).

### Early pregnancy circulating IGFBP1 independently predicts GDM

**Figure 2:**
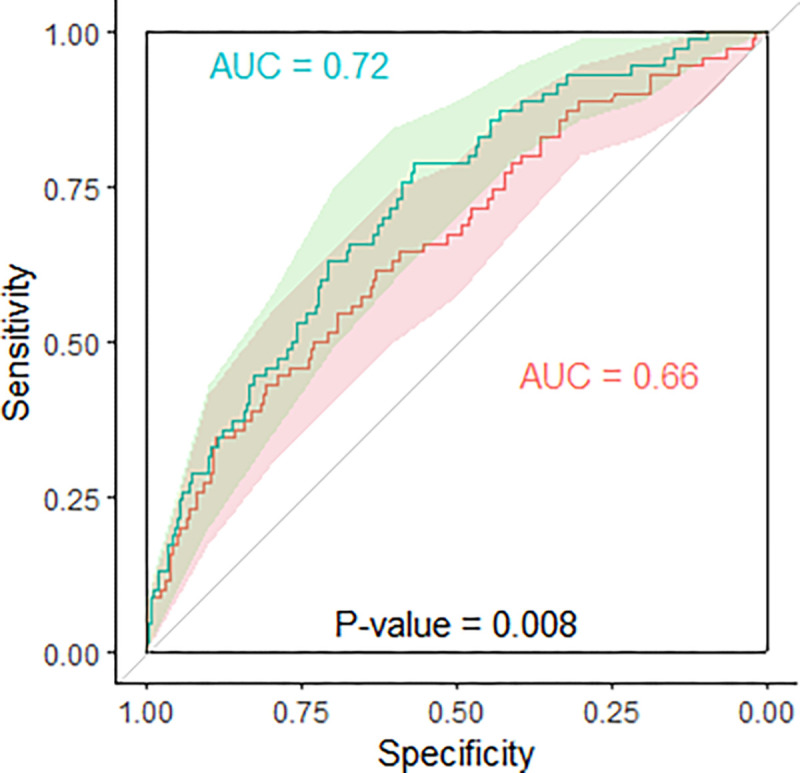
Prediction of GDM diagnosed at a median 26 weeks’ gestation from 1 st trimester plasma IGFBP1 levels in 837 Gen3G participants (70 GDM cases) Red line (clinical variables only) = maternal age, gravidity, family history of diabetes, gestational age at V1, and maternal BMI at V1. Green line: all clinical variables plus plasma IGFBP1 levels (measured a median of 9 weeks of gestation). GDM diagnosed by International Association of the Diabetes in Pregnancy Study Groups (IADPSG) criteria. Shaded area: 95% CI for each curve (2000 stratified bootstrap). Comparing Area under the curve (AUC) values with and without plasma IGFBP1 (box-cox transformation): *P*-value = 0.008

We tested whether plasma IGFBP1 measured in early pregnancy (median 9 weeks’ gestation) predicts GDM (diagnosed with International Association of the Diabetes and Pregnancy Study Groups (IADPSG) criteria applied to a 75g-OGTT at a median of 26 weeks’ gestation) in Gen3G participants (n = 837), independent of known clinical risk factors. Overall, 70 participants (8.4%) developed GDM (Supp. Table 3). Early pregnancy IGFBP1 levels alone predicted risk of incident GDM with a modest receiver operating characteristic (ROC) AUC value of 0.64. A model including only clinical variables (maternal age, gravidity, family history of diabetes, maternal BMI, gestational week at blood sampling) without IGFBP1 levels yielded ROC AUC of 0.66 (Fig. 2). A model with the same clinical variables but also incorporating early pregnancy IGFBP1 levels improved predictive ability (Fig. 2, ROC AUC = 0.72 compared to 0.66; *P* = 0.008). Using a logistic regression model, one SD increase in plasma IGFBP1 levels in early pregnancy was associated with a greater than 50% reduction in the risk for GDM in Gen3G (OR = 0.44; IQR = 0.30–0.64; *P* < 0.001; adjusted for maternal age, gravidity, gestational age at plasma IGFBP1 measurements, and maternal BMI; see Table 3).

We replicated the predictive association between early pregnancy IGFBP1 levels and GDM incidence in a nested case-control study in the MOMS cohort (n = 55 GDM cases, diagnosed based on Carpenter-Coustan criteria at a median of 29 weeks’ gestation; matched 1:2 with non-cases): the OR was 0.40 (95% CI: 0.24–0.67; *P* < 0.001, adjusted for maternal age and BMI) per SD increase in plasma IGFBP1 (measured at a median of 17 weeks’ gestation). In the SPRING cohort, we combined all GDM cases (n = 44, diagnosed in early pregnancy or at 24–32 weeks’ gestation based on IADPSG criteria) and observed an OR of 0.75 (95% CI: 0.46–1.25; *P* = 0.28; adjusted for maternal age, BMI, and gestational age at blood samples) for each SD increase in plasma IGFBP1 measured in the first trimester (median = 13 weeks’ gestation).

### Circulating IGFBP1 variations during pregnancy by GDM physiologic subtype

**Figure 3:**
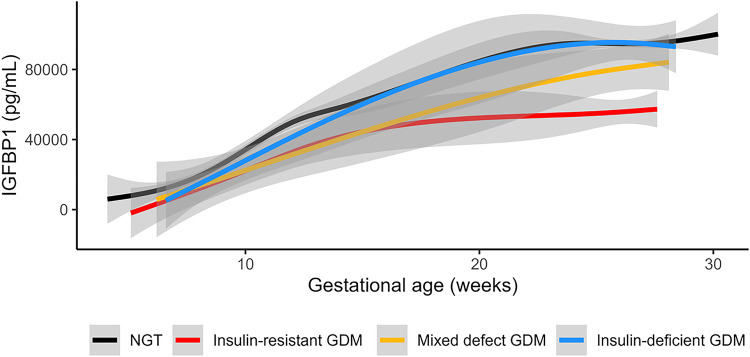
Longitudinal changes in plasma IGFBP1 levels across pregnancy in GDM subtypes and in normal glucose tolerant participants in Gen3G Abbreviations: normal glucose tolerance (NGT); Gestational Diabetes Mellitus (GDM). Sample size in each group: NGT n=767; Insulin-resistant GDM n=34; Insulin-deficient GDM n=19; Mixed defect GDM n=12.

We have previously documented pathophysiologic heterogeneity underlying GDM, demonstrating that GDM cases with a predominant insulin sensitivity defect (insulin-resistant GDM) are more likely to experience GDM-related complications at birth^10^, whereas those with a predominant insulin secretion defect (insulin-deficient GDM) had a similar risk to those with normal glucose tolerance (NGT). Given the strong association between plasma IGFPB1 and insulin sensitivity in pregnancy, we investigated the longitudinal changes in plasma IGFBP1 across pregnancy in different physiologic subtypes of GDM and in participants with NGT in Gen3G (Fig. 3). All GDM subtypes had lower mean plasma IGFBP1 levels in early pregnancy compared to the NGT group. However, the insulin-resistant GDM group had a blunted increase in IGFBP1 levels between the first and second trimester; in contrast, in those with insulin-deficient GDM, IGFBP1 levels reached similar levels to those in the NGT group during the second trimester (Fig. 3). The group who had GDM with both insulin resistance and insulin deficiency (mixed defect GDM) showed an IGFBP1 trajectory that was intermediate between the other GDM subtypes.

In Gen3G, we also found that low IGFBP1 levels in first trimester were associated with subsequent diagnosis of both insulin-resistant GDM and insulin-deficient GDM with ORs ~ 0.4 (in fully adjusted models, including maternal BMI) similar to prediction models where the outcome was all GDM (see Model 3, Table 3). However, IGFBP1 levels in the second trimester were only associated with insulin-resistant GDM (OR = 0.28 [0.16–0.47] per SD increase in IGFBP1 levels; *P* < 0.001); there was no significant association between second trimester IGFBP1 plasma levels and insulin-deficient GDM (Table 3).

## Discussion

In this study, using genome-wide RNA-seq of placental tissue, we identify *IGFBP1* as a key placental transcript associated with insulin sensitivity in human pregnancy. Our findings implicate IGFBP1 deficiency in GDM pathophysiology. We show that circulating IGFBP1 levels rise during pregnancy and are much higher in pregnancy than in the non-pregnant state, supporting the contribution of placental *IGFBP1* to elevated circulating IGFBP1 in pregnancy. In three independent pregnancy cohorts, we demonstrate a strong and consistent correlation between higher circulating IGFBP1 and greater insulin sensitivity (lesser insulin resistance), uncovering a potential compensatory mechanism in euglycemic pregnancy. Moreover, we show that low plasma IGFBP1 levels in the first trimester of pregnancy predict the later diagnosis of GDM, independent of maternal clinical risk factors (including BMI). Finally, we note that the normal pregnancy rise in IGFBP1 levels is attenuated in insulin-resistant GDM, suggesting that a defect in placental IGFBP1 release may contribute specifically to this GDM physiologic subtype.

In placental tissues, *IGFPB1* expression has previously been detected in decidual cells and in fetal placental macrophages or Hofbauer cells^21^, however, there is limited knowledge of *IGFBP1* regulation and actions in pregnancy. In in-vitro experiments in decidualized human endometrial stromal cells, *IGFBP1* was regulated by cAMP, progesterone and relaxin^22,23^ – the latter two being critical hormones for establishment and maintenance of a pregnancy^24^. Outside of pregnancy, *IGFBP1* is almost exclusively expressed by the liver^17^ and its production is regulated by insulin which inhibits its gene transcription in hepatocytes^25^. Our observations that plasma IGFBP1 levels decline after a plasma insulin rise in response to an oral glucose load introduce the possibility that insulin may downregulate the production and/or release of IGFBP1 from the placenta, similarly to the downregulation observed in hepatocytes^25^. It is also possible that other insulin sensitivity endocrine factor such as adiponectin regulates *IGFBP1* expression in placental cells^26^.

Functional studies suggest that IGFBP1 binds IGF-1 and IGF-2 with equal affinity and can either inhibit or enhance IGF actions, depending on the context^20^. In post-natal life, IGF-1 is the main active growth factor and is essential for normal growth during childhood and adolescence; while during fetal development, both IGF-1 and IGF-2 are key regulators of fetal growth^20^. Outside of pregnancy, IGF-1 enhances insulin sensitivity by suppressing hepatic glucose production^27,28^ and promoting glucose uptake in peripheral tissues^29,30^. *IGF2* is a highly expressed imprinted gene which is a key regulator of fetal growth in mammals^31^. In a recent study, pregnant mice with an *IGF2* deletion specific to placental endocrine cells did not develop the normal insulin resistance of pregnancy and gave birth to fetuses that were growth restricted and hypoglycemic^32^. In general, IGFs have higher affinity for IGFBPs than for cellular IGF-receptors, and thus, IGFBPs often act as inhibitors of biological activity^22^. However, they may also function as a circulating pool of IGFs by prolonging their half-lives and creating IGF reservoirs^18,20^. In addition, IGFBP1 has putative IGF-independent effects, and may activate PI3K/AKT signaling pathways involved in post-receptor insulin signaling directly^33^. In line with this, in vivo injection of an active IGFBP1 peptide improved insulin sensitivity in a diet-induced obesity mouse model^34^. These multiple mechanisms of action might explain some of the inconsistencies from previous animal studies attempting to establish the effects of IGFBP1 on glucose regulation^35–37^.

None of these prior studies provide insights on the specific role that IGFBP1 may have in the context of pregnancy, when there are high circulating levels of IGFs, which are suspected to influence glucose metabolism^20,32^. We speculate that placental release of IGFBP1 may regulate insulin sensitivity in pregnancy – via direct and/or indirect effects – physiologically contributing to homeostatic mechanisms to balance maternal and fetal nutrient needs. An alternative explanation is that low levels of IGFBP1 in GDM are a consequence of hyperinsulinemia with another upstream cause, but this would not be in line with the rise of circulating IGFBP1 throughout pregnancy (which is characterized by hyperinsulinemia that increases as well). In the context of GDM pathophysiology, based on our findings in individuals with insulin-resistant GDM, we speculate that the placenta may be unable to produce increasing amounts of IGFBP1 as pregnancy progresses; this deficiency in circulating IGFBP1 could contribute to excessive insulin resistance, and thus to maternal hyperglycemia detected in the late second trimester in this GDM subtype. In individuals with insulin-deficient GDM, IGFBP1 levels were low in the first trimester but were at similar concentration to levels in those without GDM at the end of the second trimester, suggesting that other pathophysiologic factors contribute to hyperglycemia in this GDM subtype. Given the differences in IGFBP1 in different GDM subtypes and increasing recognition in the field that GDM is a heterogeneous condition^38^, our finding of persistently lower IGFBP1 levels in the second trimester of pregnancies affected by insulin-resistant GDM may have implications for GDM precision medicine^39,40^. Our findings suggest that in insulin-resistant GDM, the placenta does not increase IGFBP1 production sufficiently; if this association is demonstrated to be causal, this opens the door to a novel therapeutic target for this GDM subtype. Beyond GDM, the association between lower circulating IGFBP1 levels and higher birth weight is in line with similar observations in prior report^41^ and suggests a potential explanation for the greater risk of large-for-gestational-age birthweight that we previously observed in insulin-resistant GDM^10^.

Accurately predicting GDM incidence based on early pregnancy markers could allow development and implementation of interventions to prevent GDM and its complications. However, most predictive models that rely on established clinical risk factors perform poorly^42,43^, and thus, there has been a search for reliable and replicable biomarkers. We found that low levels of circulating IGFBP1 in early pregnancy predict later diagnosis of GDM in a large population-based cohort (Gen3G), with external replication and consistent effect sizes in a separate cohort (MOMS). The effect size was more modest and not statistically significant in a cohort study of participants who all had GDM risk factors (SPRING); these inclusion criteria may have diminished the predictive ability of circulating IGFBP1 in this population. Previous studies have been inconsistent with regard to circulating IGFBP1 as a predictive biomarker for GDM, with only one study reporting on IGFPB1 levels measured before 20 weeks of gestation^44^. Our ROC analyses showed that circulating IGFBP1 levels in early pregnancy have a predictive ability beyond that of established GDM risk factors (including maternal BMI and family history of diabetes), however, the moderate ROC AUC value in a model that included IGFBP1 levels along with these clinical factors suggests that additional biomarkers would be necessary for clinical utility.

### Strengths and limitations

Our investigation has several strengths. We included a large number of placentas in our expression profiling, used transcriptome-wide RNA-seq, and leveraged an agnostic approach to implicate genes and their products in insulin sensitivity during pregnancy. Furthermore, we examined not only placental expression of *IGFBP1*, but also circulating IGFBP1 levels in three pregnancy cohorts. Our analyses included measurement of circulating IGFBP1 levels over a longitudinal timeframe that spanned both pregnancy and postpartum. In addition, we used an OGTT-based measure of insulin sensitivity that has been validated against euglycemic clamps in pregnancy. Our study also had some limitations. Although we had a large overall sample size, the number of GDM cases was somewhat modest, and the sample size for each GDM physiologic subtype was even more limited. Although we were able to tie placental expression and circulating IGFBP1 levels to detailed physiologic phenotyping, our study was observational and thus cannot establish mechanisms or causality for the associations we observed.

## Conclusions

In this study which utilized placental gene expression profiling, we implicated IGFBP1 in insulin sensitivity during pregnancy. *IGFBP1* is highly expressed by the placenta and maternal IGFBP1 levels are markedly elevated during gestation, increasing across pregnancy and dropping substantially postpartum. Both placental and circulating IGFBP1 levels are strongly and consistently correlated with maternal insulin sensitivity. Low levels of circulating IGFBP1 in early pregnancy predict the diagnosis of GDM in the late second trimester, independent of clinical GDM risk factors in two different pregnancy cohorts. We demonstrated distinct IGFBP1 trajectories in different physiologic subtypes of GDM, with insulin-resistant GDM lacking the expected increase in circulating IGFBP1 across gestation. Future studies should address whether IGFBP1 has direct or indirect effects on tissues that regulate maternal insulin sensitivity during pregnancy. If IGFBP1 is causally implicated in gestational glycemic regulation, novel therapeutic approaches based on IGFBP1 replacement as an insulin sensitizer could be envisioned and tested for precision prevention or treatment of GDM.

## Methods

### Gen3G cohort

#### Population

Gen3G is a prospective population-based cohort which recruited pregnant women from January 2010 to June 2013 at the Centre Hospitalier Universitaire de Sherbrooke (CHUS), located in the province of Quebec (Canada). Participants were demographically representative of the greater population of the region^15^. Each study participant provided informed written consent, and the study protocols were reviewed by the ethical committees from the CHUS, and from Harvard Pilgrim Health Care Institute.

We recruited 1024 pregnant women without preexisting diabetes in the first trimester (diabetes diagnosis from self-report or biochemical screening with HbA1c ≥ 6.5%). Exclusion criteria for enrollment in the cohort were non-singleton pregnancies or regular use of medications that influence glucose regulation. We collected measurements and blood samples from mothers at a first trimester visit (V1) conducted between 5 and 16 weeks of gestation (median 9 weeks), and in the late second trimester (V2) at 24 to 30 weeks of gestation (median 26 weeks; the time of universal GDM screening). We collected placental samples in addition to data on mothers and offspring at delivery.

#### Variables collection and measurements

At V1, we collected demographic data and prior medical and obstetric history; we performed standardized anthropometric measurements. Trained research staff measured weight with a calibrated scale and height with a standardized stadiometer. We calculated first trimester BMI as weight divided by squared height (kg/m^2^). At V1, we also collected additional blood samples that were drawn during the 50g glucose challenge test (GCT, performed in 95% of participants). For the current study, we excluded participants that had a first trimester random glucose or 1h-glucose post-GCT > 10.3 mmol/L (overt hyperglycemia per national guidelines at the time) as we were interested in GDM incidence (ascertained with universal testing at 24–30 weeks).

At V2, we performed similar anthropometric measurements and questionnaires as at V1. V2 occurred at the time of the fasting 75g-OGTT, which was standard clinical practice for screening and diagnosis of GDM at the CHUS. We collected additional blood samples at the fasting, 1h, and 2h time-points of the 75g-OGTT to measure insulin at each time-point, in addition to glucose. We measured glucose levels via the hexokinase method (Roche Diagnostics; CHUS biochemistry laboratory) as soon as samples were collected. We measured insulin levels via multiplexed particle-based flow cytometric assays (Human Milliplex MAP kits; EMD Millipore) from the previously frozen plasma samples (stored at −80°C until measurement). We estimated insulin sensitivity using the Matsuda Index^45^ (using glucose and insulin values during the OGTT), as previously validated against euglycemic clamps performed in pregnancy^16^.

At delivery, we collected newborn age and sex at birth using medical records, in addition to details from the end of pregnancy and delivery complications. Trained study staff collected placentas within 30-minutes of delivery using a standardized protocol. In brief, 1-cm^3^ placental tissue sample was collected from the maternal facing side, including decidual tissue (within a 5cm radius of the corresponding location of cord insertion on the other side). Each collected sample was immediately put in RNA-Later for at least 24 hours at 4°C before storage at −80°C until RNA extraction.

### RNA Extraction, Sequencing and Quality Control

We extracted total RNA (average = 19.7 ± 7.1 μg) and checked the quality of each sample using an Aligent Bioanalyzer to determine the RNA Integrity Number (RIN; average RIN = 6.7 ± 0.8). We shipped samples (3 μg) with an RIN value ≥ 5 to the Broad Institute (Cambridge, MA, USA) for sequencing. In a second round of sample quality control at the Broad Institute (Caliper Life Sciences LabChip GX system), the RNA Quality Score (RQS) for each sample ranged from 3.3 to 7.8 (average RQS = 5.9). We submitted all samples with an RQS value of 4 or higher for RNA sequencing (N = 466). We completed library preparation with 250ng of each sample, using an automated variant of the Illumina TruSeq^™^ Stranded mRNA Sample Preparation Kit (Illumina, cat #RS-122–2103). We performed Flowcell cluster amplification and sequencing according to the manufacturer’s protocols using the Illumina HiSeq 4000, to generate 101-bp paired end reads, average of 113M total reads (range 33M to 378M) per sample.

In line with best practices and the GTEx v8 pipeline^46^, we applied STAR v2.5.3a^47^ to align FASTQ/FASTA files to the human GRCh38 reference genome, using the parameters specified at https://github.com/broadinstitute/gtex-pipeline. Duplicate reads were marked using Picard MarkDuplicates, and expression was quantified with RNASeQC v2.3.6 using the GENCODE v26 annotation^48^.

Following quantification, we applied additional quality control (QC) steps. Of the 466 samples sequenced, we excluded those with > 1% of outlier genes (> 3 times the inter quartile range (IQR) above Q3 or > 3 IQR below Q1), leaving 459 samples for our final analytical data set. Among these, we had complete data on the phenotype of interest (Matsuda index) and covariates for 434 samples. Prior to differential gene expression analysis, we removed genes with low abundance, keeping only those genes with at least a count of 6 reads and a transcript per million (TPM) values > 0.5 in a minimum of 20% of samples, as well as average mappability > 0.8. After QC, 15,202 genes remained. Prior to differential expression analysis, we performed between-sample normalization using the R statistical software package edgeR^49^, then normalized and transformed gene counts to log2 counts per million reads (CPM) using Voom from the Limma R package^50^. The Gen3G placental RNA-seq data is available on dbGAP (https://www.ncbi.nlm.nih.gov/projects/gap/cgi-bin/study.cgi?study_id=phs003151.v1.p1).

### SPRING

The Study of Pregnancy Regulation of INsulin and Glucose (SPRING) is a longitudinal cohort study of pregnant participants with risk factors for diabetes that was conducted in 2015–2021. Participants were eligible if they were at < 15 weeks’ gestation and had a history of GDM, family history of diabetes or GDM, or if they had BMI ≥ 25 kg/m^2^ and had one additional risk factor according to American Diabetes Association guidelines^51^. Participants gave informed consent and underwent a fasting 75g-OGTT at < 15 weeks’ gestation, 24–28 weeks’ gestation, and 6–12 weeks’ postpartum. The latter two study visit windows were widened to 24–32 weeks’ gestation and 6–24 weeks’ post-partum to promote participant retention (including during the COVID-19 pandemic). We measured glucose and insulin levels as previous described^19^. The Matsuda index was calculated from the glucose and insulin levels measured during the OGTT^19^. GDM was diagnosed according to IADPSG criteria applied to the OGTT at the pregnancy study visits. Most participants that met IADPSG criteria at the first visit were not retested at the second visit. Blood samples from each study visit were collected in EDTA plasma tubes, processed, and frozen at −80°C for future analysis. The study was approved by the Mass General Brigham Institutional Review Board (IRB).

### MOMS

The MGH Obstetrical Maternal Study (MOMS) was conducted from 1998–2006^52^. Participants were eligible if they were receiving prenatal care at Massachusetts General Hospital. Participants provided written informed consent and were enrolled at their first prenatal visit where they donated an extra blood sample from a clinical blood draw. A subset of participants in 2001–2006 volunteered to return to donate fasting blood and urine samples at 16–20 weeks’ gestation. Glucose and insulin levels were measured as previously described^53^. Fasting plasma samples were frozen at −80°C and stored for future analyses. At 24–28 weeks’ gestation, participants without preexisting diabetes underwent universal screening for GDM with a non-fasting 50-gram GCT. If the venous blood glucose 1 hour after the GCT was ≥ 140 mg/dl, patients were referred for a diagnostic 3-hour 100g-OGTT. For this analysis, we included individuals whose OGTT results met Carpenter-Coustan criteria for GDM (≥ 2 abnormal values). Of these participants with GDM, 55 had remaining fasting samples available for analysis. We matched control participants with normal GCT results (2 for each GDM case) on year of sample collection and gestational age at sample collection. We preferentially selected control samples on which fasting glucose and insulin had previously been measured on the sample collected at 16–20 weeks’ gestation. We calculated HOMA-2S from fasting glucose and insulin values to estimate insulin sensitivity^54^(https://www.rdm.ox.ac.uk/about/our-clinical-facilities-and-mrc-units/DTU/software/homa/download).

### Bioassays for circulating IGFBP1 (all three cohorts)

We measured circulating IGFBP1 in plasma samples from all 3 cohorts in the same laboratory using a commercially available ELISA that measures free IGFBP1 (Catalog # DGB100, R&D systems, MN). The precision for the assays were: intra-assay CVs 5.6% and inter-assay CVs of 9.5%. We measured IGFBP1 levels in a blinded fashion, and we followed protocol for measurement per manufacturer’s instructions.

### Statistical analyses

For characteristics of participants in all three cohorts, we reported normally distributed continuous variables as mean ± SD, non-normally distributed continuous variables as median and interquartile range (IQR), and categorical variables as percentages. We used a log2-transformation for Matsuda index (to approach a normal distribution) in the differential placental RNA expression analyses.

### Placental differential expression analyses (using RNA-Seq data in Gen3G)

We adjusted models for maternal age, gravidity, maternal BMI at the first trimester visit, sex of offspring, and gestational age at delivery, in addition to computed surrogate variables (SVs) to account for unmeasured sources of variability, including batch effects and cell types. We used the EstDimRMTfunction from the R package isva^55^ to estimate the number of SVs to include given the residuals from the regression of Matsuda and biological covariates from the normalized counts, which resulted in 37 SVs computed by the R package SmartSVA^56^ recommended for our processed RNAseq dataset. We used Limma^57^ to identify differentially expressed genes with log_2_ Matsuda as a continuous independent variable. We reported genes that had differential expression in relation to Matsuda with *P*-values < 1.0 × 10^− 3^.

### Circulating IGFBP1 correlation analyses

We used a box-cox transformation for plasma IGFBP1 levels in Gen3G (from MASS package^58^ in R) since it was the best way to approximate a normal distribution. We conducted analyses in SPRING and MOMS cohorts using plasma IGFBP1 levels without transformation, given distributions that were relatively normal. We used Pearson correlations between circulating IGFBP1 levels and Mastuda index (log-transformed) in all three cohorts; we used partial correlations to assess the associations while taking into account maternal age, gestational age at blood draw, and maternal BMI. In Gen3G, we also used Pearson correlations to assess associations between plasma IGFBP1 (box-cox transformed) and maternal metabolic markers, as well as newborn anthropometry (transformed if needed).

### Circulating IGFBP1 and risk of GDM analyses

We conducted logistic regression analyses with the levels of circulating IGFBP1 as the independent variable and GDM as the dependent variable in Gen3G and SPRING; in MOMS, due to the matched case-control design of GDM cases to controls, we used conditional logistic regression. In Gen3G and SPRING, we used international criteria (IADPSG)^59^ to ascertain GDM, while in MOMS, we used the Carpenter-Coustan criteria^60^. In Gen3G, we additionally sub-classified GDM by the insulin physiology defect driving hyperglycemia (insulin-resistant GDM, insulin-deficient GDM or mixed defect GDM, as previously described^10^). We first built unadjusted logistic regression analyses (Model 1). We adjusted for maternal characteristics (maternal age, gravidity, gestational age at plasma samples) in Model 2, and additionally adjusted for maternal BMI in Model 3. We calculated profiled log-likelihood confidence intervals along with likelihood ratio test p-values (using MASS^58^ and glmglrt (https://CRAN.R-project.org/package=glmglrt) packages^47^ in R). In SPRING and MOMS cohorts, we employed similar modeling strategies using maximum likelihood dichotomous logistic models.

We conducted GDM predictive analyses using Receiver Operating Characteristic (ROC) curves in Gen3G to compare the predictive ability of first trimester (V1) plasma IGFBP1 levels in addition to commonly measured GDM clinical risk factors (maternal age, gravidity, family history of diabetes, gestational age at V1, and maternal BMI at V1). We compared the ROC AUC values using all the clinical factors with and without first trimester (V1) plasma IGFBP1 levels (after Box-Cox transformation). We compared the ROC AUC values from nested models using DeLong’s test using the “roc.test” function from the pROC package in R^61^. We considered differences between AUC values to be statistically significant if *P* < 0.05.

In Gen3G, we performed analyses using R version 4.3.0 (https://www.R-project.org), STATA, and SPSS version 28 only for partial correlations. In SPRING and MOMS, we performed analyses using Stata/IC version 16.1.

## Figures and Tables

**Table 1:** Characteristics of Gen3G participants included in the placental RNA sequencing analyses (N = 434)

Characteristics	Mean (SD) or Median [IQR] or N (%)
Maternal age (years)	28.7 (4.4)
Primigravid	154 (35.5%)
Gestational age at first trimester visit (weeks)	9.4 [8.1–11.6]
Maternal BMI at first trimester visit (kg/m^2^)	23.8 [21.4—27.9]
Gestational age at second trimester visit (weeks)	26.3 [25.9—27.3]
Gestational Diabetes Mellitus*	35 (8.1%)
Insulin sensitivity (Matsuda index)	6.74 [4.70—9.36]
Gestational age at delivery (weeks)	39.6 [38.7–40.3]
Fetal sex (female)	202 (46.5%)

BMI = body mass index; Matsuda calculated at the 2nd trimester visit;

* by International Association of the Diabetes in Pregnancy Study Groups (IADPSG) criteria

**Table 2:** Cross-sectional correlations between plasma IGFBP1 levels and insulin sensitivity during and after pregnancy in the Gen3G, SPRING, and MOMS cohorts

Cohort			Unadjusted Correlations		Partial correlations Adjusted for maternal age, gestational age at blood draw		Partial correlations Adjusted for maternal age, gestational age at blood draw, and maternal BMI	
	Timing	N	Corr	*P*-values	Corr	*P*-values	Corr	*P*-values
Gen3G	24–30 weeks’ gestation	816	r = 0.50	< 0.001	r=0.50	< 0.001	r = 0.35	< 0.001
SPRING	7–15 weeks’ gestation	156	r = 0.50	< 0.001	r = 0.49	< 0.001	r = 0.35	< 0.001
	24–32 weeks’ gestation	119	r = 0.55	< 0.001	r=0.55	< 0.001	r = 0.34	< 0.001
	6–24 weeks postpartum	107	r = 0.57	< 0.001	r= 0.58	< 0.001	r = 0.48	< 0.001
MOMS	16–20 weeks’ gestation	97	r = 0.60	< 0.001	r= 0.59	< 0.001	r = 0.45	< 0.001

Gen3G: Pearson correlations using second trimester plasma IGFBP1 (box-cox transformation) and Matsuda (log transformation); maternal BMI measured at 1st trimester

SPRING: Pearson correlations between plasma IGFBP1 and Matsuda (log transformation) cross-sectionally at each visit; partial correlations for post-partum visit adjusted for number of weeks postpartum (instead of gestational age)

MOMS: Pearson correlations between plasma IGFBP1 and HOMA-IS (log transformation); MOMS participants include GDM cases matched to non-GDM participants (matched on gestational age and year of sample collection)

**Table 3:** First and second trimester levels of plasma IGFBP1 (per SD increase) and risk of GDM overall and by GDM subtype in Gen3G (OR [95% CI] from logistic regressions)

	GDM (all)	Insulin-resistant GDM	Insulin-deficient GDM
Number of GDM cases/ non-GDM	N = 70/ 767	N = 34/ 767	N = 19/767
First trimester plasma IGFBP1 levels			
Model 1 (unadjusted)	OR = 0.597	OR = 0.488	OR = 0.676
	[0.460–0.768]	[0.336–0.697]	[0.416–1.079]
	*P = 5.2 x10^− 5^*	*P = 6.4x10^− 5^*	*P = 0.10*
Model 2 (adjusted)	OR = 0.388	OR = 0.285	OR = 0.617
	[0.271–0.548]	[0.170–0.464]	[0.326–1.140]
	*P = 3.4 x10^− 8^*	*P = 2.0x10^− 7^*	*P = 0.12*
Model 3 (BMI adjusted)	OR = 0.441	OR = 0.449	OR = 0.427
	[0.299–0.642]	[0.256–0.769]	[0.208–0.850]
	*P = 1.4 x10^− 5^*	*P = 0.003*	*P = 0.02*
Second trimester plasma IGFBP1 levels			
Model 1 (unadjusted)	OR = 0.452	OR = 0.216	OR = 0.992
	[0.345–0.586]	[0.128–0.340]	[0.626–1.605]
	*P = 5.6 x10^− 10^*	*P = 4.9 x10^− 14^*	*P = 0.97*
Model 2 (adjusted)	OR = 0.454	OR = 0.214	OR = 0.993
	[0.345–0.590]	[0.126–0.340]	[0.622–1.620]
	*P = 9.2 x10^− 10^*	*P = 5.8 x10^− 14^*	*P = 0.98*
Model 3 (BMI adjusted)	OR = 0.478	OR = 0.283	OR = 0.720
	[0.349–0.647]	[0.159–0.470]	[0.410–1.274]
	*P = 1.1 x10^− 6^*	*P = 1.6 x10^− 7^*	*P = 0.26*

Footnote: plasma IGFBP1 levels transformed using box-cox for optimal normal distribution, then translated in z-score for the logistic regression analyses. OR and [95% CI] are per 1 SD increase of plasma IGFBP1

Model 2: adjusted for maternal age, gravidity, gestational age at plasma samples (V1 and V2 respectively)

Model 3: Model 2 covariates + maternal BMI measured at V1

GDM diagnosis was made at a median of 26 weeks’ gestation
